# Comorbidities in people living with HIV: An epidemiologic and economic analysis using a claims database in France

**DOI:** 10.1371/journal.pone.0243529

**Published:** 2020-12-17

**Authors:** Valérie Pourcher, Julie Gourmelen, Isabelle Bureau, Stéphane Bouee

**Affiliations:** 1 Service de Maladies Infectieuses et Tropicales, Assistance Publique Hôpitaux de Paris, Hôpital Pitié-Salpêtrière, Sorbonne Université, Paris, France; 2 INSERM UMS 011, Villejuif, France; 3 Real World Evidence, CEMKA, Bourg La Reine, France; URCEco Ile de France Hopital de l'Hotel Dieu, FRANCE

## Abstract

**Objectives:**

As people living with HIV (PLHIV) age, the burden of non-HIV related comorbidities increases resulting in additional healthcare costs. The present study aimed to describe the profile, the prevalence and the incremental costs of non-HIV related comorbidities in PLHIV compared to non-HIV matched controls (1:2 ratio) in France.

**Methods:**

The French permanent sample of health beneficiaries (*Echantillon généraliste de bénéficiaires* [EGB]), a claims database representative of the national population, was used to assess comorbidities in PLHIV which were identified by the ICD-10 diagnosis codes of hospitalization, full healthcare coverage, and drug reimbursements between 2011 and 2014. The control group was matched by year of birth, gender, region of residence, and economic status. Total costs of outpatient care and hospitalizations were analysed from a societal perspective. A general linear model was used to assess the incremental cost per patient in PLHIV.

**Results:**

A total of 1,091 PLHIV and 2,181 matched controls were identified with a mean ± standard deviation age of 46.7 ± 11.5 years. The prevalence of alcohol abuse (5.8% vs 3.1%; p<0.001), chronic renal disease (1.2% vs 0.3%; p = 0.003), cardiovascular disease (7.4% vs 5.1%; p = 0.009), dyslipidaemia (22% vs 15.9%; p<0.001), hepatitis B (3.8% vs 0.1%; p<0.001) and hepatitis C (12.5% vs 0.6%; p<0.001) was significantly higher in PLHIV compared with non-HIV controls. Other comorbidities such as anaemia, malnutrition, psychiatric diseases, and neoplasms were also more prevalent in PLHIV. Hospitalizations were significantly increased in PLHIV compared to controls (33.2% vs 16%; p<0.001). Mean total cost was 6 times higher for PLHIV compared to controls and 4 times higher after excluding antiretroviral drugs (9,952€ vs. 2,593€; p<0.001). Higher costs per person in PLHIV were significantly associated to aging (42€ per patient/year), chronic cardiovascular disease (3,003€), hepatitis C (6,705€), metastatic carcinoma (6,880€) and moderate or severe liver disease (6,299€).

**Conclusion:**

Our results demonstrated an increase in non-HIV related comorbidities among PLHIV compared to matched controls. This study contributes to raise awareness on the burden of chronic comorbidities.

## Introduction

Access to effective antiretroviral therapy (ART) has significantly reduced global HIV-related morbidity and mortality and has improved life expectancy of people living with HIV (PLHIV) [1, 2], allowing the proportion of patients over 50 years to increase [3, 4]. PLHIV aging prolongs exposure to HIV and ART, while increasing the prevalence and burden of non-HIV related comorbidities [5]. Cardiovascular diseases, hypertension, diabetes, chronic renal diseases and osteoporotic bone fractures are frequent non-HIV related comorbidities in PLHIV [6–11]. Among these comorbidities, cardiovascular diseases, chronic kidney disease and osteoporosis were found to be more prevalent in PLHIV compared to non-HIV populations in high-income countries [12–19]. The increased number of age-related comorbidities occurring at an earlier age compared to non-HIV infected people suggests premature aging in PLHIV [12, 14], possibly due to HIV infection and side effects of ART. Indeed, long-term use of antiretroviral drugs, sustained HIV-associated immune activation and chronic inflammation have all been reported to be associated with an increased risk of comorbidities, including cardiovascular disease [20–22].

Non-infectious comorbidities account for more than half of deaths in PLHIV, led by cardiovascular diseases and cancer [10, 23, 24]. The evolving burden of non-infectious comorbidities in PLHIV, besides resulting in poor health outcomes, increases the complexity of managing HIV infection along with other comorbidities. Additionally, higher comorbidity burden increases costs and requires more healthcare resources [25, 26], especially in patients older than 50 years old [27]. Understanding the implication of comorbidities and cost in PLHIV may thus help to optimize care, including the choice of ART regimen and the appropriate management of comorbidities [28].

In France, between 155,000 and 205,000 people are estimated to live with HIV, and 49% were 50 years or older in 2015 [29]. However, there are scarce data on the comorbidity profiles in French PLHIV, as well as their impact on total healthcare costs. Therefore, we conducted a cross-sectional analysis using a claims database, representative of the French population; first, to identify and describe the most prevalent non-HIV related comorbidities in PLHIV including cardiovascular diseases; second, to compare the prevalence of comorbidities, healthcare resources and costs between PLHIV and matched non-HIV controls; and finally to assess the incremental cost per patient for non-HIV related comorbidities in PLHIV.

## Materials and methods

A retrospective analysis was conducted from 2011 to 2014 using the permanent beneficiaries sample (*Echantillon Généraliste de Bénéficiaires* [EGB]), a claims database representative of the French population and described in prior research [30–32]. The EGB covers 1/97th random sample of hospitals and claims database with nearly 600,000 salaried workers and their relatives and accounting for 77% of the total population. This database is accurately representative of the French population in terms of age, gender, and means reimbursed healthcare expenditures [32]. It contains a single anonymous information on age, gender, place of residence, date of death, full healthcare coverage for a long-term illness (Affection de Longue Durée, [ALD], reimbursed care in private and public hospitals, drugs and devices’ delivery dates, laboratory and radiology tests, consultations, and hospital discharge, along with the ICD-10 related diagnosis and diagnosis-group [32].

### Population

Adults aged 18 years and older were selected from the EGB database in 2011. Each HIV was matched with 2 non-HIV controls (1:2 ratio) on age, gender, universal coverage, and place of residence. All patients were followed until December 31st, 2014. PLHIV were selected with an algorithm described in prior research [33]. The criteria to identify HIV patients using this algorithm were: a full healthcare coverage for HIV, HIV-related hospitalization (ICD-10 codes: B20, B21, B22, B23, B24, F02.4, Z20.6, Z21), ART reimbursement, HIV-related biology test (i.e., genotypic resistance testing to antiretrovirals by sequencing the reverse transcriptase gene and the viral protease gene, or genotypic resistance testing to antiretroviral drugs by sequencing the envelope gene).

The control group was non-HIV subjects without any HIV-related hospitalisation, ART reimbursement or full healthcare coverage for HIV until 2014. Controls were matched to PLHIV (2:1 ratio), by year of birth, gender, region of residence in 2011, and Universal Medical Coverage (CMUc), with 100% coverage reflecting a low economic status.

### Ethical considerations

Data analysed in this study were retrieved from an anonymous database, therefore, an ethics committee’s approval was not required.

### Data collection

Demographic characteristics, comorbidities and cardiovascular risk factors were extracted from the EGB database. Antiretroviral treatments were collected from the first date of delivery until 2011. Clinical characteristics of patients were described including duration of full healthcare coverage for HIV, number of hospitalizations between 2011 and 2014 for an infection (ICD-10 code of the main diagnoses beginning with A or B), and Charlson comorbidity index at the end of the study. This index predicts the one-year mortality for a patient who may have a range of comorbid conditions and was calculated and modified by the algorithm provided by Quan et al in 2011 [34]. This calculation was based on diagnosis in the hospital discharge database during the study period.

### Comorbidities

Comorbidities were identified by hospitalization-related diagnosis using the ICD-10 code, full healthcare coverage, or reimbursements for a specific drug. CVD were defined as having or having had a cardiovascular condition and were represented by chronic ischemic heart disease, stroke, peripheral artery disease (PAD), chronic rheumatic heart diseases, cardiac failure, pulmonary embolism and thrombophlebitis, stroke or transient ischemic attack (TIA), unstable angina and/or myocardial infarction. These conditions were defined by prior hospitalizations and ALD full coverage. Prior hospitalizations, ALD full coverage, and specific treatments were then analysed to identify cardiovascular risk factors such as hypertension, dyslipidaemia, alcohol abuse, and diabetes. It was essential to report CVD and risk factors since they are increase in aging PLHIV. Bone fractures were identified from a prior hospitalization using the ICD-10 code. However, some ART regimens are associated with increased rate of bone fractures due to loss of bone mineral density. Hepatitis B and C were determined by the ALD full coverage, ICD-10 codes B182 from a previous hospitalization, or treatment of these conditions. Besides the aging-related comorbidities of interest, the most prevalent comorbidities in PLHIV compared to controls were represented by depressive disorders; neurotic, stress-related and somatoform disorders; disorders due to psychoactive substance consumption; nutritional deficiency, anaemia, and neoplasms. These prevalent comorbidities were presented separately in this analysis.

### Healthcare resource utilization and costs

Healthcare resources (general practitioners and specialists’ consultations) and direct costs from a societal perspective (*i*.*e*., paid in euros by patients) were reported for PLHIV and controls in 2014 or in the last 12 months before death from 2011 to 2014. Healthcare resources utilization (HCRU) and costs were obtained from separate data and related to hospitalizations, consultations, medications, laboratory tests, nursing, medical transportation, and other expenses. Medications costs were reported separately for ART and other treatments. Based on the reported costs, an algorithm was used to categorize the costs related to medications, consultations, etc. The mean total cost per patient was calculated by adding all reported costs in the database.

### Statistical analysis

Continuous data were presented as mean values ± standard deviation (SD), and categorical data as frequency counts and percentages (%). Qualitative variables (comorbidities and healthcare resource utilization) were compared between PLHIV and controls using the Chi-square test. Yates continuity correction or Fisher's exact test were used when sample sizes were less than 5.

Comparisons of comorbidities between PLHIV and controls were assessed for the comorbidities and risk factors of interest. The most prevalent comorbidities in PLHIV were compared to those of controls, and significant differences were presented.

Healthcare costs were compared between PLHIV and controls using the Wilcoxon non-parametric test.

A generalized linear model (GLM) using gamma distribution with a log-link function was used to analyse the relationship between total healthcare costs (excluding the cost of ART) and comorbidities and to assess how the comorbidities might result in an incremental cost per patient. Based on their likelihood of impacting healthcare costs, the following variables were included in the model: age, gender, low socioeconomic level (CMUc), chronic cardiovascular diseases (*i*.*e*, chronic ischemic heart disease, peripheral arterial disease, pulmonary embolism, phlebitis and thrombophlebitis), neoplasm (ICD-10 code C00-C96), chronic renal disease, hypertension, type 1 or 2 diabetes, dyslipidaemia, alcohol abuse, hepatitis B or C co-infection, metastatic solid tumor (ICD-10 code C76-C80), and non-overlapping conditions of the Charlson comorbidity index (*i*.*e*., dementia, chronic pulmonary disease, rheumatic disease, moderate or severe liver disease, paraplegia or hemiplegia). A backward selection process was used to select the most appropriate model according to the Akaike Information Criteria (AIC).

The EGB database contains approximately 650,000 subjects. The sample size was calculated according to the prevalence of AIDS in France and a preliminary power analysis. We estimated that about 1,000 subjects would be enough to identify a significant (alpha risk<5%) difference of prevalence of 5% between PLHIV and controls with a power of 80%.

All analyses were considered statistically significant for a *p*-value<0.05 and were performed using SAS© software Version 9.4 (Cary, USA).

## Results

### Population characteristics

Overall, 1,006 patients (18 years and older) had a full reimbursement coverage for HIV, 22 patients had been hospitalized for HIV, and 63 patients had been treated with an ART in 2011.

**Fig 1:**
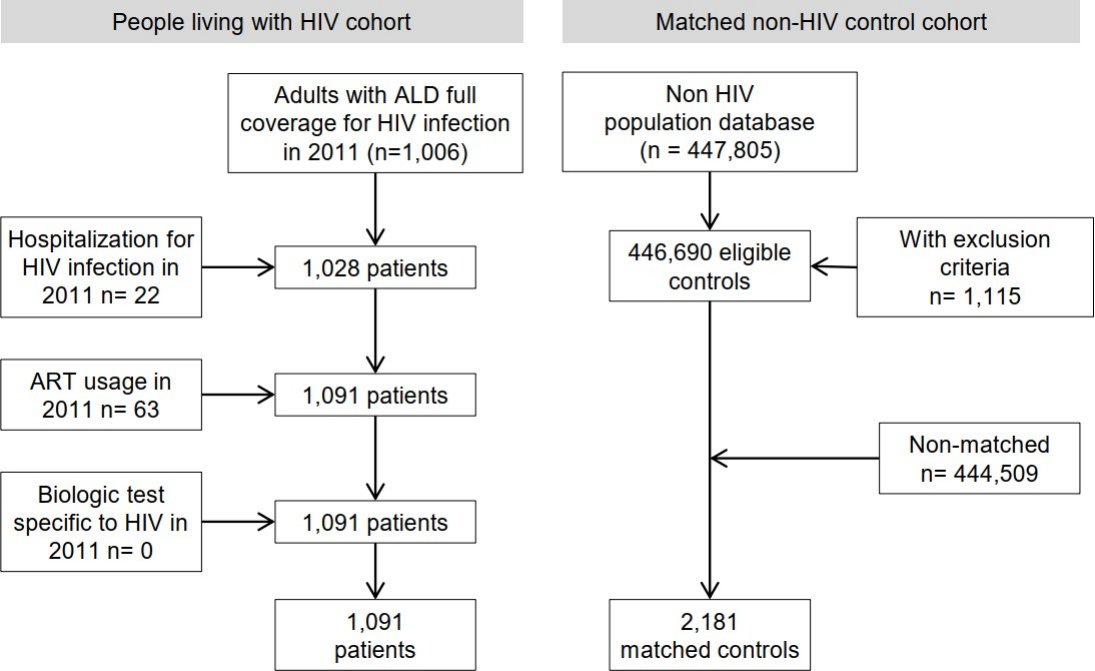
Flow chart selection of the cohorts.

Fig 1 presents the number of HIV-positive patients and matched controls. Controls were matched to cases by age, gender, and place of residence.

The characteristics of PLHIV and matched-controls for the entire sample are presented in Table 1. PLHIV were predominantly male (68.8%) and had a mean age of 46.7 years with 36.0% of patients older than 50. A low socioeconomic status (CMUc) was present in 15.5% of patients. The median duration of HIV (time since onset of full coverage for HIV) was 10 years (range: 0 to 40 years, quartile 25 and 75: 5 and 15 years, respectively).

**Table 1:** Characteristics of the study population.

	Total population		
	PLHIV (n = 1,091)	non- HIV matched controls (n = 2,181)	P value
**Gender, male, n (%)**	751 (68.8)	1,502 (68.9)	0.986
**Mean age, years (SD)**	46.7 (11.5)	46.6 (11.5)	0.986
**Age groups; n (%)**			
18–29 years	70 (6.4)	140 (6.4)	1.000
30–39 years	226 (20.7)	452 (20.7)	
40–49 years	403 (36.9)	806 (37.0)	
50–59 years	250 (22.9)	500 (22.9)	
60–69 years	101 (9.3)	201 (9.2)	
≥ 70 years	41 (3.8)	82 (3.8)	
**Low level of income (CMU coverage), n (%)**	170 (15.6)	334 (15.3)	0.841
**Time since onset of full coverage for HIV (years)**			
Mean (SD)	10.2 (6.2)		
Median (range: 0 to 40 years)	10.0		
Quantile 25 / Quantile 75	5.0 / 15.0		
**Infection-related hospitalizations (between 2011 and 2014); n (%)**			
0	966 (88.5)	2166 (99.3)	***<0*.*001***
1	96 (8.8)	13 (0.6)	
2	15 (1.4)	1 (0.0)	
≥3	14 (1.3)	1 (0.0)	
**Charlson CI; mean (SD)**	4.9 (2.6)	0.9 (1.6)	***<0*.*001***
Treatment with at least one ART in 2011	729 (66.8)	-	***-***

CI; comorbidity index, HIV: human immunodeficiency virus, PLHIV: Patient living with HIV, SD: standard deviation

A total of 2,181 controls were matched to PLHIV in a ratio of 2:1, except for one PLHIV subject for whom only one valid control was found. Among the 1,091 PLHIV, 729 (66.8%) had been treated with at least one ART in 2011 (Table 1).

### Comorbidities

#### Comorbidities of interest

The prevalence of comorbidities and risk factors of interest for the period 2011–2014 are described in Table 2. PLHIV had a significantly higher prevalence of comorbidities compared to non-HIV controls: alcohol abuse (5.8% vs. 3.1%; p<0.001), chronic or end stage renal disease (1.2% vs. 0.3%; p = 0.003), peripheral artery disease (2.5% vs. 1.4%, p = 0.020), cardiovascular diseases (chronic ischemic heart disease; cardiac failure; chronic rheumatic heart disease, history of stroke/ transient ischemic attack or peripheral artery disease; 7.4% vs. 5.1%; p = 0.009), dyslipidaemia (22.0% vs. 15.9%; p<0.001), hepatitis B (3.8% vs. 0.1%; p<0.001), and hepatitis C (12.5% vs. 0.6%; p<0.001).

**Table 2:** Prevalence of comorbidities and risk factors in PLHIV and non-HIV matched controls.

	Total population		
n (%)	PLHIV (n = 1,091)	Controls* (n = 2,181)	p-value**
**Cardiovascular risk factors**			
Hypertension	306 (28.0)	564 (25.9)	0.182
Dyslipidaemia	240 (22.0)	346 (15.9)	***<0*.*001***
Diabetes	72 (6.6)	172 (7.9)	0.187
Alcohol abuse	63 (5.8)	68 (3.1)	***<0*.*001***
**Cardiovascular diseases**			
Chronic ischemic heart disease	45 (4.1)	66 (3.0)	0.102
Peripheral artery disease	27 (2.5)	30 (1.4)	***0*.*024***
Cardiac failure	18 (1.6)	26 (1.2)	0.284
Unstable angina and/or MI	14 (1.3)	32 (1.5)	0.673
Pulmonary embolism or phlebitis and thrombophlebitis	12 (1.1)	16 (0.7)	0.284
Stroke or transient ischemic attack (TIA)	12 (1.1)	15 (0.7)	0.219
Chronic rheumatic heart diseases	3 (0.3)	3 (0.1)	0.407
Any CVD ***	81 (7.4)	112 (5.1)	***0*.*009***
**Other comorbidities**			
Bone fractures	27 (2.5)	40 (1.8)	0.222
Chronic renal disease, dialysis or renal transplant	13 (1.2)	7 (0.3)	***0*.*003***
Hepatitis B	42 (3.8)	2 (0.1)	***<0*.*001***
Hepatitis C	136 (12.5)	13 (0.6)	***<0*.*001***

* non-HIV matched controls

**unadjusted in the matched sample

*** Any CVD: chronic ischemic heart disease; cardiac failure; chronic rheumatic heart disease, history of stroke/ transient ischemic attack or peripheral artery disease

CVD; cardiovascular disease, MI: Myocardial infarction

PLHIV: People living with HIV.

#### Most prevalent comorbidities

Table 3 depicts the most prevalent comorbidities other than those of interest in PLHIV vs controls from 2011 to 2014. PLHIV had a significantly higher prevalence of depressive disorder (6.8% vs. 2.2%; p <0.001); neurotic, stress-related, and somatoform disorders (2.8% vs. 1.2%; p = 0.007); disorders due to psychoactive substance consumption (11.3% vs. 5.5%; p<0.001); nutritional deficiency (3.3% vs. 1.1%; p<0.001); anaemia (7.4% vs. 2.8%; p<0.001) as well as neoplasm (7.8% vs. 5.5%; p = 0.010). Neoplasms were also significantly more prevalent in PLHIV than controls: digestive organ (2.3% versus 0.9%; p = 0.001), lymphoid or hematopoietic system (1.8% versus 0.4%; p<0.001), connective and soft tissue (0.8% versus 0.2%; p = 0.010).

**Table 3:** Other prevalent comorbidities in PLHIV and non-HIV matched controls.

	Total population		
n (%)	PLHIV (n = 1,091)	Controls* (n = 2,181)	p-value**
**Psychiatric disorders**			
**Mental and behavioural disorders due to psychoactive substance use** (ICD-10 code F10-19)	123 (11.3)	120 (5.5)	<0.001
**Depressive disorder** (ICD-10 code F32-33)	74 (6.8)	47 (2.2)	<0.001
**Neurotic, stress-related and somatoform disorders** (ICD-10 code F40-48)	31 (2.8)	26 (1.2)	0.001
**Anaemia** (ICD-10 code D50-64)	81 (7.4)	60 (2.8)	<0.001
**Malnutrition** (ICD-10 code E40-46)	56 (5.1)	35 (1.6)	<0.001
**Nutritional deficiencies** (ICD-10 code E50-64)	36 (3.3)	24 (1.1)	<0.001
**Malignant neoplasms** (ICD-10 code C00-97)	85 (7.8)	120 (5.5)	0.010
Bone and articular cartilage	0 (0.0)	1 (0.0)	1.000
Breast	7 (0.6)	8 (0.4)	0.273
Connective and soft tissue	9 (0.8)	4 (0.2)	0.014
Digestive organs	25 (2.3)	20 (0.9)	0.002
Endocrine glands and related structures	2 (0.2)	9 (0.4)	0.355
Eye, brain and central nervous system	1 (0.1)	3 (0.1)	1.000
Lip, oral cavity and pharynx	3 (0.3)	15 (0.7)	0.132
Male genital organs	9 (0.8)	27 (1.2)	0.286
ther female genital organs	4 (0.4)	5 (0.2)	0.492
Respiratory system and intrathoracic organs	4 (0.4)	22 (1.0)	0.051
Secondary and ill-defined	16 (1.5)	19 (0.9)	0.119
Skin	7 (0.6)	17 (0.8)	0.663
Stated or presumed to be primary, of lymphoid, haematopoietic and related tissue	20 (1.8)	9 (0.4)	<0.001
Urinary organs	5 (0.5)	4 (0.2)	0.171

* non-HIV matched controls

**unadjusted in the matched sample

ICD-10: international classification of diseases 10th revision, PLHIV: patients living with human immunodeficient virus

*Mortality*. Over the four year-period (2011–2014), mortality rate was higher in PLHIV compared to controls (4.3% vs. 1.9%; p<0.001).

### Healthcare resource utilization

Healthcare resource utilization in PLHIV and non-HIV matched controls in 2014 is presented in Table 4. PLHIV were more likely to consult a general practitioner compared to controls (76.7% vs. 79.9%; *p* = 0.030). However, the mean number of consultations was higher for PLHIV than controls in those with at least one consultation, (6.2% vs. 5.0%; *p*<0.001). Outpatient specialist 8oio-//79+4 were lower for PLHIV compared to controls (58.5% vs. 63.8%; *p* = 0.004) in contrast to inpatient specialist consultations (46.7% vs. 17.2%; p<0.001). The mean number of specialist consultations was similar between the two groups. However, PLHIV were hospitalized twice more frequently than controls (33.2% vs. 16%; *p*<0.001).

**Table 4:** Healthcare resource utilization in PLHIV and non-HIV matched controls in 2014.

	Total population		
	PLHIV (n = 1,091)	Controls* (n = 2,181)	p-value**
**Outpatient (ambulatory care)**			
general practitioner consultations; n (%)	837 (76.7)	1,743 (79.9)	***0*.*035***
Mean number of consultations (SD)	6.2 (5.6)	5.0 (4.5)	***<0*.*001***
Consultations of specialists in the private sector; n (%)	638 (58.5)	1,392 (63.8)	***0*.*003***
Mean number of consultations (SD)	4.0 (5.5)	4.1 (5.0)	0.127
specialist consultations in public hospitals; n (%)	509 (46.7)	376 (17.2)	***<0*.*001***
Mean number of consultations (SD)	3.7 (4.5)	3.6 (4.5)	0.129
**In patients (hospital care)**			
Hospitalizations; n (%)	362 (33.2)	350 (16.0)	***<0*.*001***

* non- HIV matched controls

**unadjusted in the matched sample

PLHIV: People Living with HIV.

### Healthcare costs

Mean healthcare costs per patient from a societal perspective in 2014 are presented in Table 5. Overall costs were significantly increased in PLHIV compared to controls with higher costs of medications (12,578€ *vs*. 433€; *p*<0.001), majorly driven by ART (6,809.6€). The mean total healthcare cost per patient was 6 times higher for PLHIV compared to controls and remained almost 4 times higher after excluding ART cost (9,952€ *vs*. 2,593€; *p*<0.001).

**Table 5:** Mean annual costs per patient in PLHIV and non-HIV matched controls in 2014 from a societal perspective.

	PLHIV (n = 1,091)		Controls* (n = 2,181)		p-value of the means
	Mean Costs (SD), €	% of the total cost	Mean Costs (SD), €	% of the total cost	
**Cost of ambulatory care (outpatient)**	14,515.7 (25,755.3)	86.6%	1,565.7 (3,305.2)	60,4%	***<0*.*001***
Medical appointment fees	581.6 (869.8)	3.5%	428.2 (701.9)	16.5%	***<0*.*001***
Dentist appointment fees	144.0 (689.0)	0.9%	127.4 (472.5)	4.9%	***0*.*035***
Medical carers (including nurses)	279.7 (1,515.8)	1.7%	167.0 (1,038.1)	6.4%	***<0*.*001***
Laboratory tests	409.0 (394.5)	2.4%	87.8 (214.9)	3.4%	***<0*.*001***
ART	6,809.6 (5,541.7)	40.6%	0	0.0%	***<0*.*001***
Medication (excluding ART)	5,768.1 (24,954.3)	34.4%	433.1 (1,345.0)	16.7%	***<0*.*001***
Medical devices	313.8 (1,220.6)	1.9%	219.1 (661.5)	8.4%	***0*.*042***
Transport	198.1 (1,252.6)	1.2%	100.6 (1,279.8)	3.9%	***<0*.*001***
Other services	11.8 (176.2)	0.1%	2.6 (32.5)	0.1%	***<0*.*001***
**Hospitalisations (inpatient)**	2,245.8 (8,410.0)	13.4%	1,027.4 (5,645.9)	39.6%	***<0*.*001***
**Total cost (ambulatory care + hospitalisation)**	16,761.5 (28,054.3)	100%	2,593.1 (7,979.3)	100%	***<0*.*001***
**Total cost (ambulatory care + hospitalisation) excluding ART**	9,951.9 (27,353.3)	59.4%	2,593.1 (7,979.3)	100%	***<0*.*001***

* non- HIV matched controls

ART: anti-retroviral treatment, SD: standard deviation

### Incremental healthcare costs in PLHIV

The multivariate analysis of total healthcare costs in PLHIV, excluding ART cost, is presented in Table 6. Significant contributors to high incremental costs included aging (42€ per patient per year), chronic cardiovascular disease (3,003€), hepatitis C (6,705€), metastatic carcinoma (6,880€), and moderate or severe liver disease (6,299€).

**Table 6:** Multivariate analysis of total healthcare costs in PLHIV, excluding ART cost. A generalized linear model.

	Incremental cost per year (€)	Coefficient	95% CI of coefficient		P
			Lower	Upper	
**Age (per year)**	42	0.014	0.004	0.024	***0*.*003***
**Low economic status**	-624	-0.238	-0.528	0.069	0.112
**Chronic cardiovascular disease**	3,003	0.703	0.328	1.116	***0*.*001***
**Chronic pulmonary disease**	1,230	0.349	-0.151	0.923	0.199
**Chronic renal disease**	3,389	0.766	-0.095	1.947	0.133
**Hepatitis C**	6,705	1.187	0.850	1.550	***<0*.*0001***
**Neoplasm**	1,106	0.319	-0.108	0.802	0.168
**Metastatic carcinoma**	6,880	1.205	0.300	2.306	***0*.*017***
**Moderate or severe liver disease**	6,299	1.144	0.443	1.990	***0*.*003***
**Paraplegia and hemiplegia**	3,129	0.724	-0.174	1.971	0.177
**Rheumatic disease**	4,211	0.888	-0.346	2.919	0.260

CI: confidence interval

## Discussion

Our study using a claims database, representative of the French population, found a higher prevalence of cardiovascular diseases, dyslipidaemia, chronic renal disease, hepatitis B and hepatitis C, and alcohol abuse in PLHIV compared to non-HIV matched-controls.

Furthermore, the number of hospitalizations was significantly more prevalent in PLHIV. Interestingly, hypertension and dyslipidaemia were the most prevalent cardiovascular risk factors in PLHIV (28% and 22%, respectively), particularly in those over 50 years old. While hypertension and diabetes were highly prevalent in both PLHIV and controls, dyslipidaemia and alcohol abuse were more common in PLHIV, likely contributing to the higher burden of cardiovascular diseases in this population.

Previous cohort studies found a higher prevalence of cardiovascular diseases and renal failure or chronic kidney disease in PLHIV compared to non-HIV populations in Italy, Netherlands and Denmark [12–14, 19], in line with our results. HIV-infection *per se* and the long-term exposure to ART may contribute to the higher prevalence of cardiovascular diseases, by increasing the risk of developing a traditional cardiovascular risk factor (*e*.g. by worsening dyslipidaemia) and affecting the pathogenic process through effects on inflammation or endothelial function [35]. The aging of PLHIV also naturally increases the frequency of comorbidities in this population and other studies have underlined this factor [36]. In the present study, the use of an aged-matched control group removed the effect of age in the increasing rates of comorbidities. Yet, such method allows to interpret our results independently from age, gender and region of residence.

Associations between long-term exposure to ART and renal or cardiovascular adverse events have been previously reported. Abacavir and darunavir boosted with ritonavir have been linked to an increased risk of cardiovascular events including myocardial infarction [21, 22], while long-term exposure to tenofovir disoproxil fumarate, has been associated to decreased renal function and mineral bone density loss in PLHIV [37]. In our study, 57.5% of PLHIV were treated with tenofovir disoproxil fumarate, 50.2% with ritonavir boosted protease inhibitor, and 20.7% with abacavir However, further interpretation is limited, as the specific correlation between ART and comorbidity has not been addressed.

ART has also been associated with an increased risk of bone fractures [20]. In contrast with our results, Guaraldi et al. found a higher prevalence of bone fractures in 2,854 PLHIV compared to 8,562 age- gender- and race-matched controls [12]. Their study included PLHIV with a mean age comparable to ours, but the mean duration of HIV infection was longer, resulting in a possible longer exposure to ART. In our study, fractures were defined by an ICD-10 code for bone fractures during a prior hospitalization, therefore, it was not possible to determine whether these fractures were due to osteoporosis. We found a higher prevalence of comorbidities in PLHIV such as anaemia, malnutrition and nutritional deficiency, psychiatric diseases (depressive disorders, anxiety and disorders related to psychoactive substance abuse), and neoplasms. Anaemia associated with malnutrition and underweight are common HIV complications [38]. Higher rates of psychiatric illnesses, including depression and anxiety have been described in PLHIV compared to the general population [39, 40]. Cancers such as liver, anal and lung cancers as well as Hodgkin lymphoma were also found to be increased in PLHIV as compared to the general population between 1997 and 2009. Yet, the authors found a decreased incidence over the last years, and an excess of risk for patients with low CD4+ cells [41]. All these comorbidities represent additional burdens that should be considered in the management of PLHIV.

In this study, the analysis of direct annual costs from a societal perspective revealed that healthcare resource utilization and costs were significantly higher in PLHIV compared to matched controls.

The total annual cost associated to outpatient care and hospitalization was 6-fold increase in PLHIV compared to controls and 4-fold increase after excluding ART cost. In a previous French study, the per-patient annual cost of treatment in HIV-infected patients was estimated at 14,821€ in 2010 [42], which is in line with our findings. In Italy, Quiros-Roldan et al. showed that the average total per capita direct cost attributable to HIV in 2012–2014 was 10,374€ whereas the per capita cost of ART was 7,562€ [19]. Another recent study conducted in France estimated the mean cost associated with ART from the National Health Insurance perspective (public cost of the drug) at 11,400€ per patient in 2014 [43]. However, the population included in this study was not representative of French PLHIV since it was restricted to a single university hospital in Paris and patients had at least one ART prescription in the last 12 months. Moreover, costs were calculated by assuming that ART regimen were taken for 12 months with maximum adherence.

Annual cost of medications other than ART was found to be 13-fold higher in PLHIV than in matched controls (5,768€ vs 433€) and was the driver of total excess costs in PLHIV along with hospitalization costs but excluding ART (2,246€ vs 1,027€).

The proportion of subjects consulting outpatient specialists were lower for PLHIV compared to controls, in contrast with the consultations of inpatient specialists. Although some comorbidities were more frequent among PLHIV, such difference is probably mainly driven by the management of HIV infection. The complexity of the management of PLHIV, particularly with regard to drug prescriptions, may explain that hospital physicians turn to be the referent for HIV infection. They are infectious disease specialists and internists rarely working in a private practice. The more frequent private hospital-based specialist visits could also be due to the patient’s social situation. A US study showed that patients with low income and public/no health insurance were more commonly attending community-based clinics [44]. Since 15% of PLHIV had CMU coverage in our study, we believe that PLHIV would rather choose the public sectors.

PLHIV were hospitalized twice as often as controls, resulting in a two-fold increase of hospitalization costs among PLHIV. Interestingly, a recent study showed that among half of hospitalizations of PLHIV, an opportunistic infection was documented and could contribute to the two-fold increase in the hospitalization rate and in the costs of hospitalizations in PLHIV [45]. In a recent Canadian study, including patients aged 20–49 years, the annual per-patient cost of medication without ART was 3-fold higher in PLHIV compared to non-HIV controls, and the cost of hospitalisation was increased by 2.5-fold [46]. The authors also showed that renal comorbidity was associated with the highest costs, followed by bone and cardiovascular diseases. In another retrospective study using administrative health claims data in the USA in 2011–2013, Gallant et al. assessed the direct medical cost burden associated with chronic kidney disease, cardiovascular events, and fracture/osteoporosis in PLHIV treated with ART. Their results indicated that chronic kidney diseases and cardiovascular events were associated with significantly higher total all-cause healthcare costs in PLHIV compared to matched controls without a comorbid condition or event [28]. The high prevalence of comorbidities in PLHIV is associated with substantial excess costs [47].

Our study is one of the first real-world and nationwide studies comparing comorbidity patterns between PLHIV and matched non-HIV controls using a sample that better represents the French population. Our findings suggested that the high prevalence of certain comorbidities may have contributed to increased total costs in PLHIV. We have assessed the incremental cost of comorbidities, which reflect the additional burden of managing patients beyond the costs associated with HIV, and by excluding ART which is the main component of total HIV health cost [42]. In this analysis, cardiovascular and liver diseases, hepatitis C as well as metastatic carcinoma, but not chronic kidney diseases, were found to contribute mostly to the higher incremental cost per patient in PLHIV. In Italy, Quiros-Roldan et al. evaluated the association between age, gender, period of follow-up, CD4+ cell count at baseline and chronic diseases using multivariate regression models [19]. The per capita cost was positively associated with age (+36€ every year of age), cardio-cerebrovascular diseases (+2,087€), liver diseases (+4,388€) and cancer (+7,557€), in accordance with our findings. These results are in line with excess cost in the management of PLHIV with such comorbidities.

Our study has some limitations inherent to medical claims data. First, information on diagnosis was available only for patients with a full coverage for a long-term chronic illness or hospitalization. Second, the lack of clinical and laboratory data (especially CD4 levels), as well as information on other cardiovascular risk factors such as smoking status and body mass index did not allow to control on the differences in healthcare utilization and costs associated with these variables. Third, although controls were matched to PLHIV on age, sex, place of residence and economic status, differences in some demographic and lifestyle-related factors may be present, thus limiting the interpretation of our results regarding the comparison of comorbidities without adjustment of all potential confounding factors. Fourth, HCRU and cost derivation were not directly associated since costs were separately reported in the database. An effort was made to assign costs to categories including medication, consultations etc.; however, this might have limited in understanding the direct link between costs and HCRU.

To our knowledge, this is one of the first real-world nationwide studies assessing comorbidities patterns in PLHIV and matched non-HIV controls. The EGB database allows access to the nationwide claims and hospitalisation databases, which enables collection of data more representative than those of registries. At the time of the study, the available data provided information on a random sample of 77% of the French population [8]. Since the information is accurate and exhaustive and can be linked to claims and hospitalisations data, the EGB can be considered as one of the most reliable databases in France for epidemiological studies, avoiding selection and information bias [31]. Moreover, the risk of attrition can be considered as insignificant since there are very few reasons for a subject to leave one of the three main public healthcare insurance schemes.

## Conclusion

PLHIV had a significantly higher prevalence of comorbidities compared to non-HIV controls and a higher mean number of consultations. PLHIV were hospitalized twice as often as non-HIV controls and had a significantly higher mortality rate over the four-year period of the study. The mean cost was significantly higher in PLHIV compared to controls for all resources. After excluding ART, the cost of other medications and more hospitalizations contributed to a mean total healthcare cost per patient almost 4-fold higher. Significant contributors to high incremental costs included, aging, chronic cardiovascular disease, hepatitis C, metastatic carcinoma, and moderate or severe liver disease. As PLHIV age, it is crucial to provide support for the management of comorbidities to achieve both improvement in patient outcomes and reduction of healthcare resources utilization. Prospective studies are needed to contribute to reducing the burden of comorbidities and costs in PLHIV.
